# Comprehensive Utilization of Immature Honey Pomelo Fruit for the Production of Value-Added Compounds Using Novel Continuous Phase Transition Extraction Technology

**DOI:** 10.3390/biology10080815

**Published:** 2021-08-23

**Authors:** Guo Liu, Tao Hou, Shenglan Guo, Hongyu Lin, Meng Chen, Jianyin Miao, Xiaojuan Liu, Yahui Huang, Yong Cao, Yaqi Lan, Mingyue Song

**Affiliations:** 1 Guangdong Provincial Key Laboratory of Nutraceuticals and Functional Foods, College of Food Science, South China Agricultural University, Guangzhou 510642, China; liuguo@scau.edu.cn (G.L.); ht.taohou@gmail.com (T.H.); guo_llt@163.com (S.G.); u3567752@connect.hku.hk (H.L.); miaojy8181@scau.edu.cn (J.M.); liuxj@scau.edu.cn (X.L.); caoyong2181@scau.edu.cn (Y.C.); 2 College of Horticulture, South China Agricultural University, Guangzhou 510642, China; yahuihuangzz@scau.edu.cn; 3 Laboratory and Equipment Administration Department, Guangdong University of Petrochemical Technology, Maoming 525000, China; Julia-chan2014@hotmail.com

**Keywords:** immature pomelo fruit, sequential extraction, value-added compounds, continuous phase transition extraction under low temperature

## Abstract

**Simple Summary:**

For the first time, this study investigated the extraction of bioactive substances with different polarities from immature honey pomelo fruit (IPF), a by-product of pomelo planting processing that causes resource waste and environmental pollution, using novel continuous phase transition extraction technology (CPTE). The results showed that CPTE was suitable for extracting essential oil, naringin, and pectin in sequence according to their polarities. The naringin extraction process was optimized by the response surface methodology, resulting in an extract ratio up to 99.47%. Moreover, the pectin extracted from IPF by CPTE showed better quality compared to commercial counterparts, as evidenced by lower protein and ash contents and higher white value. Together, these results suggested that CPTE could be a promising technology to improve the application value of IPF. For instance, the extracted bioactive components can be utilized as nutraceutical food ingredients. The scientific insights from this study will contribute to the development of functional food ingredients and comprehensive utilization of farming by-products.

**Abstract:**

The immature honey pomelo fruit (IPF) is a huge agro-industrial by-product generated during pomelo planting. Although IPF is rich in nutrients, more than 95% of IPF is discarded annually, which causes resource waste and a serious environmental problem. Here, we report a novel continuous phase transition extraction technology (CPTE) to improve the comprehensive utilization of IPF by sequentially generating high value products and solve pollution problems related to their disposal. First, essential oil was successively extracted by CPTE at a yield of 1.12 ± 0.36%, in which 43 species were identified. Second, naringin extraction parameters were optimized using the response surface methodology (RSM), resulting in a maximum extraction rate of 99.47 ± 0.15%. Finally, pectin was extracted at a yield of 20.23 ± 0.66%, which is similar to the contents of commercial pectin. In conclusion, this study suggested that IPF was an excellent potential substrate for the production of value-added components by CPTE.

## 1. Introduction

Pomelo (*Citrus grandis* (L.) *Osbeck*), a member of the citrus family, is widely cultivated in Southeast Asia and some other Asian countries [1,2,3]. During the processing of pomelo planting, massive amounts of IPF are generated as a primary by-product or waste, which accounts for 40–50% of the total fruit quantity [4]. Although a small part of IPF is utilized to produce traditional Chinese medicine [5], the majority of IPF is disposed directly, resulting in a waste of resources and risk of environmental pollution. In recent years, studies have reported that flavonoids [6,7] and naringin [8] could be extracted from immature pomelo dropped fruits. Pomelo peel, as a pomelo waste, was used to extract bioactive compounds [9,10] or to an prepare adsorbent for improving environmental pollution [11].

It has been reported that IPF is rich in nutrients and various active ingredients, including essential oil, naringin, pectin, limonin analog, etc. Moreover, the antioxidant, anti-inflammatory, and anti-carcinogenic activities grant significant health and medicinal value to IPF [12,13]. Effective reuse of these IPF components will provide considerable economic value. Naringin, as the main high-value component, has been limited in its application due to poor water solubility. Alkaline water extraction is the common method used in naringin industrial extraction due to lower levels of impurities [14]. However, the extensive use of acids and base in the conventional method is hindered by the amount of environmental pollution it generates, making it a less attractive option for the future industrial production of naringin. Therefore, an environmentally friendly, efficient, and automatic extraction technology is favored. In addition, traditional extraction methods usually produce only one class of active substance from IPF, and there are still a variety of high value components remaining.

**Figure 1:**
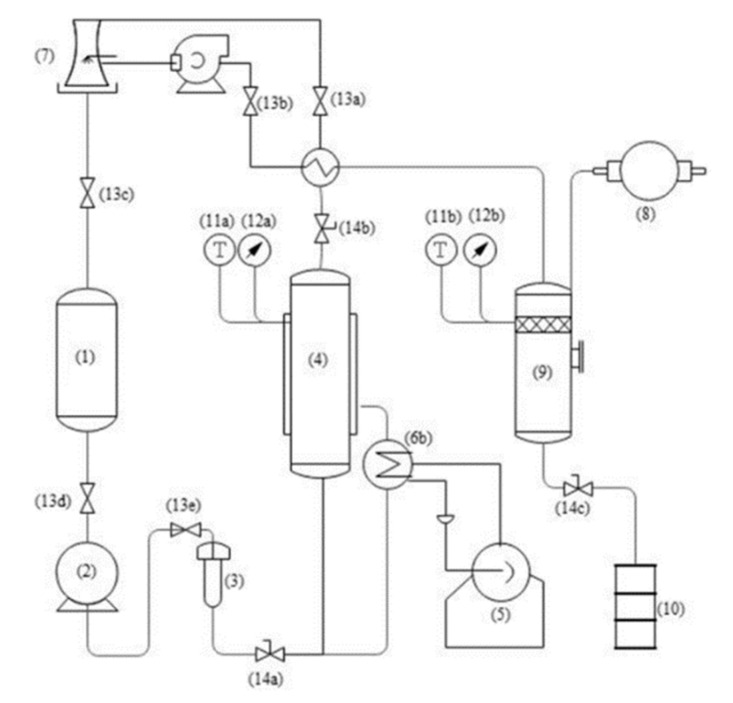
Schematic diagram of CPTE extraction (A) (1. Solvent bank, 2. Pressure pump, 3. Pre-heater, 4. Extraction kettle, 5. Boiler, 6. Heat exchanger, 7. Cooling tower, 8. Vacuum pump, 9. Desortion tank, 10. Sample collector, 11. Temperature measuring device, 12. Pressure measuring device, 13. One-way valve, 14. Control valve). Briefly, sample was filled into the extraction tank (4). Under the pressure of the pressure pump (3). The solvent is first heated to the desired temperature through a heat exchanger (6) and then flowed into extraction kettle (4) for extraction. The extract went into the desortion tank (9), target extract substance flowed into sample collector (10), the condensed solvent flowed back into solvent bank (1) to extract again.

Therefore, we have developed CPTE technology for the intensive processing of IPF. Compared to the conventional technologies, CPTE can be used for the extraction of various natural products with different polarity by altering the extraction pressure and temperature, which will contribute to a wider application [15]. Furthermore, the solvent used in CPTE can be recycled, and the extraction process is continuous, together resulting in higher efficiency and lower cost. A schematic diagram is shown in Figure 1. With these advantages, it has been successfully applied in the comprehensive utilization of soy sauce by-products [15]. In this study, soybean oil and the isoflavone in soy sauce residues were extracted through successive steps. The results showed that not only could the oil and isoflavone be extracted efficiently, but the quality is much better due to the lower extraction temperature compared to that obtained by traditional methods. CPTE has been also applied in tangerine peel oil extraction from the dried tangerine peels [16]. To date, the CPTE system has been successively developed at both the pilot and commercial scale.

Therefore, the preparation of economically important products from IPF using CPTE will significantly improve the added value of IPF, increase economic benefits, decrease waste treatment costs, and reduce environmental pollution. In this study, we successively obtained essential oil from IPF using CPTE and analyzed its composition. We then optimized the CPTE parameters for the extraction of naringin from de-fatted IPF using RSM. Additionally, we acquired pectin from IPF residues after essential oil and naringin extraction and characterized its physicochemical properties.

## 2. Materials and Methods

### 2.1. Materials and Chemicals

Dried immature honey pomelo fruits were supplied by Li pomelo Guangdong Agricultural Science and Technology Co., Ltd. (Meizhou, China) and were stored at room temperature. The composition of the IPF (protein, sugar, essential oil, naringin, pectin, and ash) was analyzed according to methods adapted from previous research [17,18,19,20,21].

HPLC-grade acetonitrile and hexane were purchased from Fisher Scientific (Springfield, NJ, USA). n-butane (99.99%) was obtained from Shenzheng Shenyan Gas Co., Ltd. (Shenzhen, China). Galacturonic acid and the naringin standard were purchased from Aladdin Co., Ltd. (Shanghai, China). Anhydrous methanol, phosphoric acid, and petroleum ether were available from Guangzhou Chemical Reagent Factory (Guangzhou, China).

### 2.2. Sample Collection

IPF was mainly produced in April and May each year, so the IPF samples were collected in both April and May. Two samples were washed twice with tap water, then cut in half and dried in oven at 35 °C until its weight remained constant. The dried IPF samples were stored at room temperature for further analysis.

### 2.3. Measurements of Pectin and Naringin

The essential oil content was determined by the Soxhlet extraction method [22]. The pectin content was determined using the phenol-Carbazole method with galacturonic acid as the standard [17]. The naringin content was quantified using high performance liquid chromatography (HPLC) [23] with minor modifications. Specifically, samples were loaded onto a Shimadzu HPLC (15C) equipped with a PDA detector and separated using a mobile phase composed of (A) deionized water containing 0.2% phosphoric acid and (B) acetonitrile containing 0.2% phosphoric acid. Elution was carried out using a gradient of 25% B over 20 min at a flow rate of 1.0 mL/min at 25 °C. The HPLC system was equipped with a YMC-Pack Pro C18 column (4.6 mm × 250 mm, 5 μm) at 283 nm.

### 2.4. Essential Oil Preparation and Composition

Prior to extraction, the IPF was ground into a 40 mesh powder. The essential oil was extracted under n-butane using CPTE equipment. The extraction was performed under 0.5 MPa for 60 min at 50 °C. The solvent was concentrated at 70 °C for 70 min.

The extracted essential oil was then analyzed with a gas chromatography mass spectrometer (GC-MS) (7890A-5975C, Agilent, Santa Clara, CA, USA) equipped with a DB-5 capillary column (30 m × 0.25 mm, 0.25 μm) following a published method [24]. The temperature was programmed as follows: 50 °C for 2 min, then a gradual increase to 130 °C at a rate of 8 °C/min, followed by an increase to 250 °C at a rate of 5 °C/min. Then, the temperature was held at 250 °C for 8 min. High purity helium was used as the carrier gas at a flow rate of 1 mL/min. Using a syringe, the injection of diluted sample (1 g essential oil diluted in 10 mL n-hexane) was 1 μL at 250 °C. The split ratio was 1/50. The MS was operating in the electron impact mode (70 eV) at an interface temperature of 200 °C and the ion source temperature of 250 °C. The chemical composition of the essential oil was identified by matching the mass spectra to mass spectral libraries (NIST08.L). The concentration of the identified compound was calculated based on the percentage of the relative peak area (%).

### 2.5. Naringin Extraction Optimization

In order to improve the extraction rate of naringin, all extraction experiments were performed in accordance with a central composite design (CCD) with 3 factors and 3 levels based on preliminary experiments. These factors were extraction temperature, time, and raw material granularity (RMG), respectively. The factors and independent variables are listed in Table 1. Statistical significance of the fitted second order quadratic model equations were tested using ANOVA. Statistical analysis was performed using Design-Expert version10.0.0 software (Stat-Ease, MN, USA).

The remaining IPF after essential oil extraction was used as the starting material for naringin extraction. Anhydrous methanol was used extract naringin under 0.3 MPa. The solvent was concentrated at 75 °C. The extracted naringin and the naringin remaining in IPF dregs after extraction were analysis by HPLC, using the parameters described above. The naringin extraction rate was calculated using the following equation:

(1) $$\mathrm{Extraction}\;\mathrm{rate}\;\left(\%\right)=100-100\times \frac{\mathrm{the}\;\mathrm{naringin}\;\mathrm{in}\;\mathrm{IPF}\;\mathrm{dregs}\;\left(g\right)}{\mathrm{the}\;\mathrm{naringin}\;\mathrm{in}\;\mathrm{defatted}\;\mathrm{IPF}\;\left(g\right)}$$

### 2.6. Pectin Extraction and Physicochemical Property

The remaining IPF dregs after naringin extraction were used as the starting materials for pectin extraction. Extractions were conducted under continuous deionized water (Ph = 1.5) under 0.3 MPa at 90 °C for 60 min. The solvent was concentrated by vacuum evaporation at 90 °C for 60 min. Pectin concentrate was desalinated using a small electrodialysis desalination unit (HMTECH-1220, Hangzhou Huamo Technology Ltd., Hangzhou, China). Subsequently, 2 volumes of 95% ethanol were added to the sample and incubated for 4 h under 4 °C to precipitate pectin. The pectin was separated by high speed centrifugation, then washed three times with 95% ethanol followed by freeze-drying to obtain the final product. The pectin yield was calculated using the following equation:

(2) $$\mathrm{Pectin}\;\mathrm{yield}\;\left(\%\right)=100\times \frac{\;\mathrm{the}\;\mathrm{weight}\;\mathrm{of}\;\mathrm{extracted}\;\mathrm{pectin}\;\left(g\right)}{\mathrm{the}\;\mathrm{weight}\;\mathrm{of}\;\mathrm{IPF}\;\mathrm{dregs}\;\mathrm{after}\;\mathrm{extraction}\;\mathrm{naringin}\;\left(g\right)}$$

The extracted pectin was analyzed for the determination of the content of galacturonic acid, moisture content, ash content, degree of esterification (DE), protein content, and total sugar content, according to methods adapted from previous researches [17,18,19,20,21]. The analysis grade pectin obtained from Sigma Chemical Co. (St. Louis, MO, USA) was employed as a reference substance.

### 2.7. Statistical Analysis

All assays were performed in triplicate, and the results are expressed as the mean ± standard deviation (SD). Student’s *t*-test was performed to test the mean difference between two groups, whereas analysis of variance (ANOVA) followed by Duncan’s multiple range test was used for the comparison of differences among three or more groups. *p <* 0.05 was used as the threshold for determining significance.

## 3. Results and Discussion

### 3.1. Samples Composition Analysis

**Figure 2:**
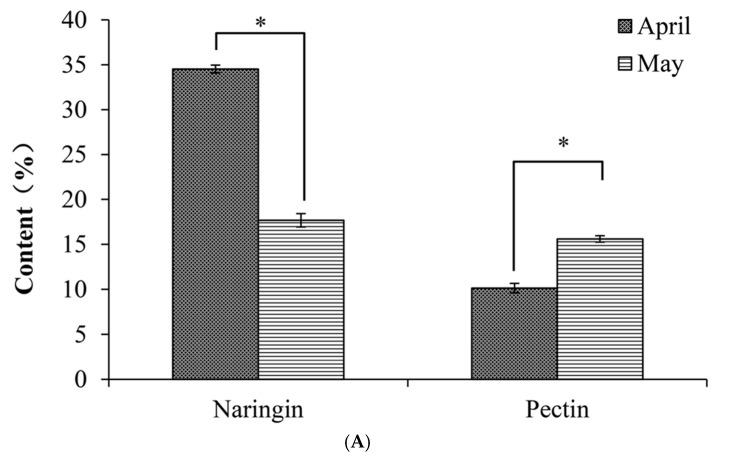

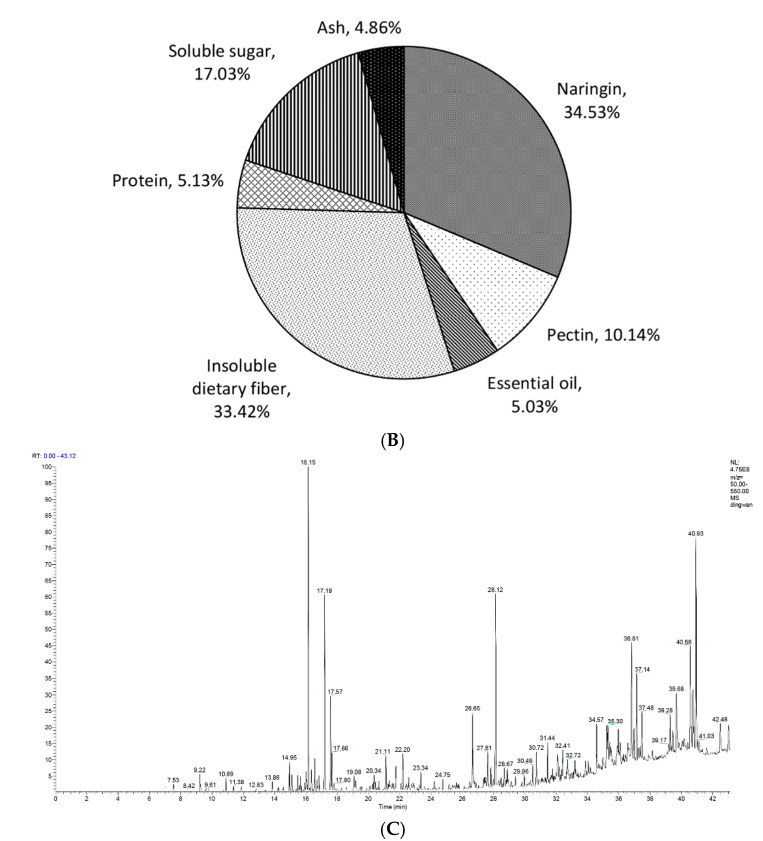
(**A**) Naringin and pectin content in immature honey pomelo fruit in April and May, respectively; (**B**) The composition of immature honey pomelo fruit in April; (**C**) Chromatogram (GC-MS) of IPF essential oil. Results were presented as the mean ± SD (*n* = 3). The asterisks in the bar chart indicate statistical significance between groups according to Student’s *t*-test (*p <* 0.05).

In order to determine which sample should be used for further analysis, the content (dry weight, DW) of naringin and pectin of IPF produced in April and May were quantified (Figure 2A). We found that the naringin content of IPF in April was dramatically higher than that of IPF in May, at 34.53 ± 0.74% and 17.67 ± 0.43%, respectively (*p <* 0.05). This is in agreement with previous research showing that flavonoid content is the highest during the immature period [25]. However, the pectin content was significantly lower in April than in May, (10.14 ± 0.36% vs. 15.60 ± 0.53%, *p <* 0.05). As the naringin content in April was twice as high as in May, we selected the IPF collected in April for the further study, even though the pectin content was slightly lower.

Then we determined the levels of essential oil and other components in IPF harvested in April. As shown in Figure 2B, the level of essential oil was only for 5.03% in IPF. Markedly, naringin was found to be the most abundant component, accounting for up to 34.53%. The protein content and total sugar content were 5.13% and 17.03%, respectively. The ash level was 4.86%. Other components such as insoluble dietary fiber constituted 33.42% of IPF. In summary, the essential oil, naringin, and pectin, which were three economically important components that can be widely utilized, accounted for 49.69% of IPF dry weight in total, indicating that the IPF in April was a valuable raw material.

### 3.2. Essential Oil Preparation and Composition

Essential oil, as a volatile oil, is a general term for oily liquids in plants which have an aromatic odor. Many studies have shown that pomelo peel essential oil presents numerous biological activities, including antibacterial, anti-inflammatory, as well as swelling and pain relief effects [26,27]. The essential oil in IPF was extracted using CPTE technology, ultimately producing a 1.12% ± 0.36% yield. Additionally, the composition of the extracted essential oil was analyzed. The GC-MS chromatogram of essential oil is shown in Figure 2C and the composition of essential oil is displayed in Table 1. A total of 43 species were identified in essential oil isolated from IPF, which accounted for 93.71% of the essential oil component. Particularly, there were up to 10 kinds of alcohol compounds (22.42%), six kinds of terpene compounds (17.81%), three kinds of ketone compounds (18.64%), eight kinds of ester compounds (15.55%), and six kinds of alkane compounds (8.01%). Importantly, D-limonene was the highest single substance (10.24%), followed by osthole (8.69%), 2,4-di-tert-butylphenol (7.29%), trans-α, α-5-trimethyl-5-ethylene Base tetrahydro-2-furan methanol (6.44%), and camphor (6.41%).

D-limonene is a common substance in citrus essential oils and a major contributor to citrus aromas. It is considered as a marker substance in essential oils, such as pomelo, orange peel, and bergamot [28], and is found at a level that exceeds 80% in pomelo peel oil [29]. Moreover, recent studies have reported the antibacterial and antioxidative activities of D-limonene [30]. Osthole, the second most abundant substance in IPF essential oil, is a coumarin compound with strong pharmacological activity. It has been used to treat cardiovascular, endocrine, and immune diseases [31,32,33]. Moreover, 2,4-di-tert-butylphenol, which makes up 7.29% of IPF essential oil, is currently used as an intermediate of light stabilizers and antioxidants. It also exhibits strong insecticidal effects [34,35]. Trans-α, α-5-trimethyl-5-ethylene Base tetrahydro-2-furan methanol, which accounts for 6.44% in IPF essential oil, is commonly used in daily fragrances and in the preparation of lavender and geranium essential oils. It has also been detected in Ilex hainanensis [36]. Camphor, found at a level of 6.41% in IPF essential oil, is fractionated from the volatile oil of plants belonging to the polygonaceae family and shows insecticidal, anti-inflammatory, cooling, warming, and pain-relieving effects [37]. Therefore, these components are crucial due to their contributions to both aromatic properties and bioactive functions of IPF essential oil.

In a previous study, a microwave irradiation treatment was conducted to extract essential oil from pomelo, and it performed better than hydrodistillation in terms of the yield of essential oil (1.88 ± 0.06% yield) [38]. In another previous study, pomelo peel oil was extracted by steam distillation, cold pressing, and mechanical dermabrasion. The yields were 1.68 ± 0.07%, 0.82 ± 0.09%, and 0.61 ± 0.21%, with 41, 42, and 60 species detected in the oil, respectively [39]. The steam distillation method has been shown to be inefficient and consume high levels of energy in the extraction of essential oils. Cold press and mechanical dermabrasion are simple methods that produce high quality oil. However, they both have obvious disadvantages, including inefficiency and high cost. In contrast, the CPTE technology we developed offers efficient and low-temperature extraction, which is suitable for the mass production of essential oil and can overcome the disadvantages seen with other traditional extraction methods. Currently, CPTE technology has been widely applied in the extraction of tangerine peel oil, fish oil, bergamot essential oil, tea oil, and other types of oils [40].

### 3.3. Model Fitting of Naringin Extraction Optimization

The IPF residue after essential oil extraction was used as the starting material for naringin extraction, and the naringin extraction rates obtained under different conditions are listed in Table 2. A good fit of the response and variables was obtained by multiple regression. The second-order polynomial equation is shown below:

(3) $$\begin{array}{ll} R_{1}=99.55+0.19 & \times \;A+0.47\times \;B-0.0005\times \;C-0.21\times \;A\times B\\ \; & -0.076\times \;A\times C+0.37\times \;B\times C-0.47\times A^{2}-0.82\times {\;B}^{2}\\ \; & -0.81\times {\;C}^{2} \end{array}$$

ANOVA analysis revealed that the polynomial model was statistically significant (*p* = 0.0003 < 0.001) (Table 3). The p-value was 0.2375 in relation to lack-of-fit (*p >* 0.05), indicating that the error of the test is small, and the non-test factor has little influence on the result. The goodness of fit (R^2^ = 0.9646) illustrated that the model has a high degree of fit and reflects real experimental results.

### 3.4. RSM Analysis of Naringin Extraction

**Figure 3:**
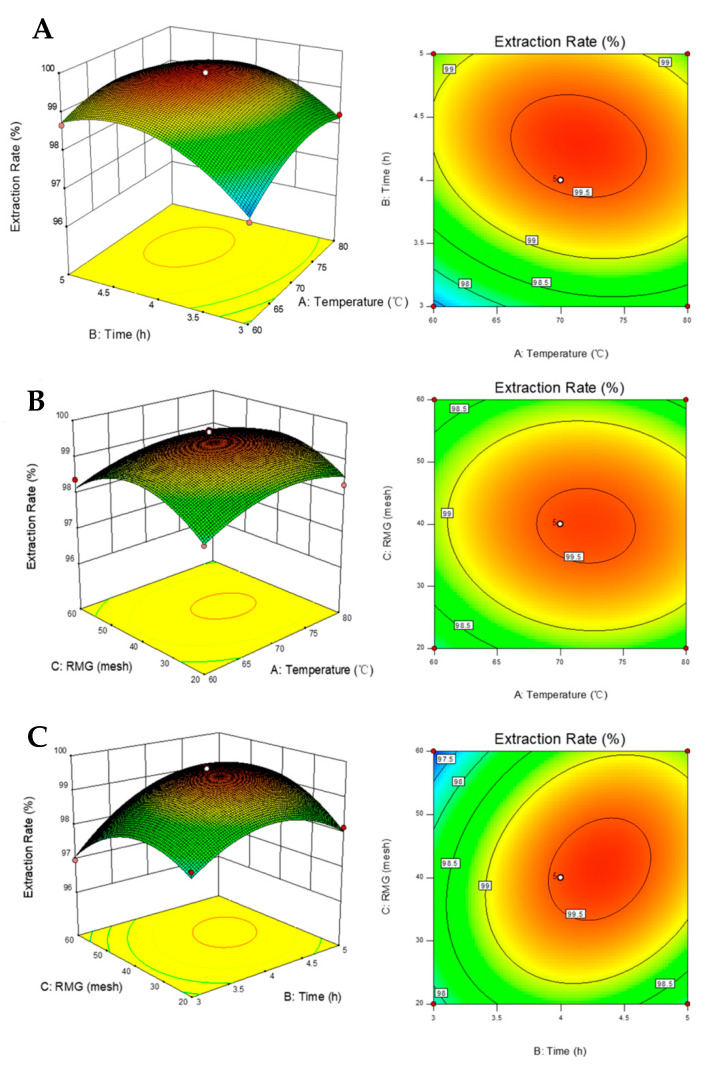
Three-dimensional response surface graphs and contour plots for the effects on naringin extraction rate under the following parameters: (**A**) temperature and time; (**B**) RMG and temperature; and (**C**) time and RGM.

The relationship between independent variables and their effects on the extraction rate of naringin were determined using a three-dimensional response surface curve generated by modeling (Figure 3).

We found that all parameters tested had significant effects on the extraction process. Noticeably, ANOVA analysis revealed that the extraction time is the most important factor affecting the extraction rate, followed by temperature. As the temperature rose, the extraction rate of naringin also increased, particularly at the beginning of the extraction process. In addition, our results indicated that the solubility of naringin was greatly affected by temperature. Moreover, an increased temperature could reduce the viscosity of the solvent and improve solute vapor pressure [41], leading to the increased efficiency of naringin extraction. However, temperature increases beyond a certain extent would change the solvent into a gaseous state, resulting in inefficient extraction. Our analysis also revealed an impact of the material to solvent ratio on the extraction rate. Over a longer extraction period, the response surface increased continuously, but was less pronounced towards the end of the extraction process. The possible reason for this phenomenon was that, at the beginning of the extraction process, the naringin content was relatively higher in defatted IPF, so the extraction was easier and more efficient. As the process continued, the level of naringin in defatted IPF gradually decreased and the impurities in defatted IPF competed with naringin [42]. For RMG, when the mesh was increased from 20 to 60, the response showed an early growth, followed by a decrease. When the RMG was below 40 mesh, the contact area between the solvent and the material was insufficient, resulting in inefficient extraction. In contrast, when the materials were larger than 40 mesh, the extraction rate of naringin decreased with increasing mesh count. This is likely due to the excessive fineness of the powder material, which led to an increase in the bulk density of the material in the extraction vessel under the action of extraction solvent and pressure [43]. Thus, it affected the normal flow rate of the extraction, and even led to blockage of the extraction pipeline.

### 3.5. Model Verification and Naringin HPLC Analysis

The biological activities of naringin have been shown in many studies. In the medical and food industries, naringin is the subject of significant demand as a pharmaceutical component and food additive. The traditional extraction methods for naringin still have the disadvantages of low purity, low extraction rate, and environmental pollution. According to our analysis using Design-Expert software, the optimum conditions for maximum naringin extraction rate were 76.4 °C for 3.7 h at 39.1 mesh RMG. The predicted extraction rate was 99.32%. We then verified this result by extracting the naringin under the optimum conditions and determining the corresponding extraction rate. For our implementation, the conditions were adjusted to 75 °C for 4 h extraction time at 40 mesh RMG. The test was performed in triplicate and the actual extraction rate was 99.47 ± 0.15%, which was very close to the predicted value for the optimal conditions.

**Figure 4:**
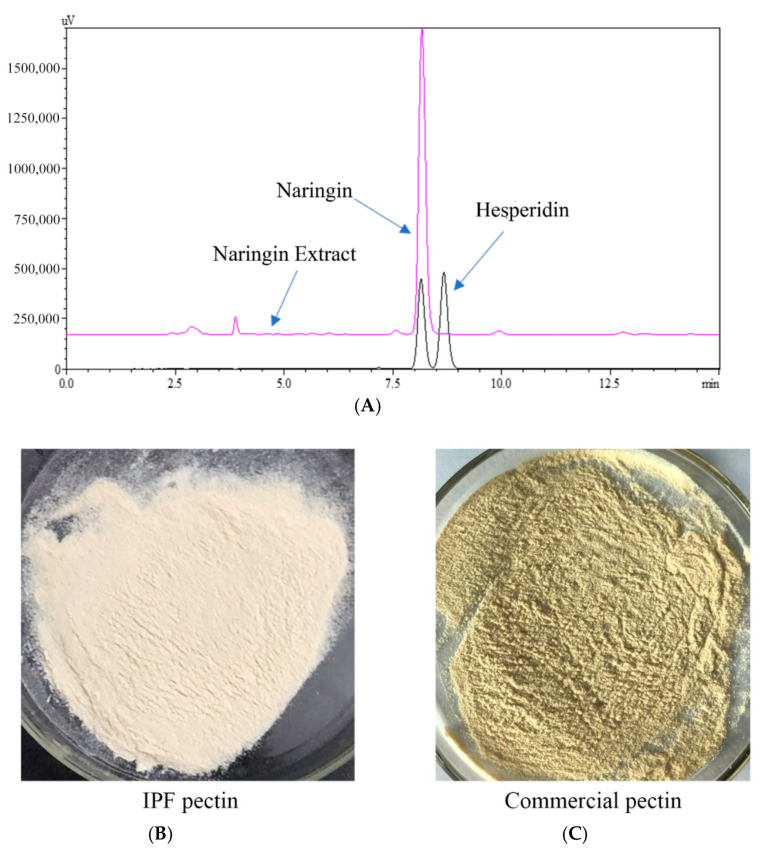
HPLC chromatogram of naringin extract (**A**); Freeze-dried IPF pectin (**B**) and purchased pectin (**C**).

Next, we compared the extracted sample and commercially available standards by HPLC. As shown in Figure 4A, according to the standards, the retention times of naringin and hesperidin were 8.20 and 8.80 min, respectively. Further, the HPLC chromatogram of extracted sample showed a clear primary peak at the retention time of 8.20 min, suggesting that naringin was the major component of the extracted sample. Hesperidin was not detected in the sample. Furthermore, there were some small peaks in the chromatogram of the extracted sample, representing some impurities that need to be identified in future studies. The naringin purity (DW) in the extracted sample was 61.21 ± 2.30% calculated by the standard curve. Altogether, these results indicated that high purity naringin could be obtained from IPF by CPTE, which offers the advantage of efficiency, continuity, and cyclical extraction. The solubility of naringin in water is only 0.1% at room temperature, so it has poor extraction efficiency in a traditional solid-liquid extraction process. Some researchers [44] have optimized the conditions for extracting flavonoids from pomelo peel using 20–100% ethanol solution. This process required about 4–8 h of operation time, which limited its application. In a separate study, the effects of ethanol concentration, temperature, and time on total flavonoid extraction from yuzu peels were investigated [23]. The purity of obtained naringin was about 24.49%, indicating that, although the efficiency was improved by optimizing the extraction, the resulting naringin required further purification. Additional studies showed that naringin could be further purified from 3.92% to 38.07% using macroporous resin [45]. Currently, alkaline water is commonly used as the solvent in naringin extraction, causing low efficiency and high energy consumption. Notably, the most impactful disadvantage of alkaline water-based extraction is environmental pollution. In this study, favorable pressure and solvent conditions were used to obtain high purity naringin extract from IPF by CPTE technology, demonstrating an easier and more efficient purification process. As the solvent can be collected and recycled, this novel extraction is able to avoid polluting the environment, which allows for the efficient utilization of IPF in industrial production.

### 3.6. Pectin Extraction and Physicochemical Properties

After naringin extracting, the IPF dregs remaining were used as the starting material for further pectin extraction. We found that the IPF dregs contained 44.02 ± 1.30% pectin, which was 3.12 times higher than that of the original proportion. This indicated that the extraction of essential oil and naringin from the IPF during the process led to the increase of the percentage of pectin remaining in IPF dregs, which would promote the effective extraction of pectin [46].

After the extraction process described earlier, pectin was obtained from IPF dregs with a yield of 20.23 ± 0.66%. Data in Table 4 show the basic ingredients and content of IPF pectin, and a comparison between IPF pectin and commercial pectin (Sigma-Adrich, St. Louis, MI, USA). The total sugar and galacturonic acid content in IPF pectin was significantly lower than that of commercial pectin, indicating the presence of potential small molecule carbohydrate impurities in IPF [47], which might be because IPF pectin was extracted only once, and no repurification process was performed. Additionally, both the protein and ash content of IPF pectin was dramatically lower than commercial pectin. Interestingly, the color of IPF pectin was obviously whiter (higher white value) than that of commercial pectin as shown in Figure 4B. This might be attributed to the extraction of essential oil and naringin, during which the butane and methanol removed most of the low and medium polarity impurities such as pigments [48]. Moreover, the degree of esterification (DE) of IPF pectin and commercial pectin were 44.65 ± 0.02% and 30.45 ± 0.02%, respectively, indicating that they were both low DE pectin. In industry, most of the pectin extracted from citrus is high DE pectin, and low DE pectin can be obtained by acid treatment extraction processes. The low DE pectin obtained by CPTE without acid treatment might be a result of the vacuum and high-pressure environment during the extraction process [49], which could affect the physicochemical properties of IPF pectin. Altogether, our results demonstrate that pectin extraction following essential oil and naringin by CPTE is a more convenient, efficient, and environmentally friendly method.

## 4. Conclusions

In this study, IPF has been used to produce value-added components (including essential oil, naringin and pectin) via a novel CPTE technology. The naringin extraction process was optimized using RSM, and the extract ratio of naringin was up to 99.47%. The quality of extracted IPE pectin, characterized by protein content, ash content, and white values, were much better than commercial pectin. Together, these results indicate that CPTE is an efficient and eco-friendly extraction technology that provides a significant advancement in the development and utilization of IPF and offers new insights pertaining to the comprehensive utilization of this agro-industrial by-product.

## Figures and Tables

**Table 1:** Chemical composition of IPF essential oil.

Peaks	Time/Min	Compound	Formula	Molecular Weight	Relative Content/%
1	9.22	Picolinic acid	C_6_H_5_NO_2_	123.11	0.63
2	14.95	*β*-Pinene	C_10_H_16_	136.23	0.97
3	16.01	1-Isopropyl-2-Methyl-Benzene	C_10_H_14_	134.22	0.89
4	16.15	(R)-(+)-Limonene	C_10_H_16_	136.23	10.24
5	16.35	3,7-Dimethyl-1,3,6-octatriene	C_10_H_16_	136.23	0.66
6	16.56	n-Hendecane	C_11_H_24_	156.31	1.17
7	17.19	trans-α, α-5-trimethyl-5-ethylene Base tetrahydro-2-furan methanol	C_10_H_18_O_2_	170.25	6.44
8	17.58	2-[(2R,5S)-5-Methyl-5-vinyltetrahydro- 2-furanyl]-2-propanol	C_10_H_18_O_2_	170.25	2.98
9	17.66	Linalool	C_10_H_18_O	154.25	1.26
10	19.07	Benzoic acid	C_7_H_6_O_2_	122.12	0.69
11	21.11	3,4-Dimethylbenzaldehyde	C_9_H_10_O	134.18	1.27
12	21.75	Hexadecane	C_16_H_34_	226.44	1.56
13	22.20	Dodecane,2,6,11-trimethyl-	C_15_H_32_	212.41	1.19
14	26.65	Bicyclo [7.2.0]undec-4-ene,4,11,11- trimethyl-8-methylene-, (1R,4Z,9S)-	C_15_H_24_	204.35	3.20
15	27.82	Cedrene	C_15_H_24_	204.35	0.73
16	28.12	2,4-Di-tert-butylphenol	C_14_H_22_O	206.32	7.29
17	30.49	Spathulenol	C_15_H_24_O	220.00	0.86
18	30.72	Caryophyllene oxide	C_15_H_24_O	220.35	1.35
19	32.08	Heptadecane	C_17_H_36_	240.47	2.69
20	32.72	4(15),5,10(14)-Germacratrien-1-ol	C_15_H_24_O	220.00	1.35
21	33.19	2,6,11,15-Tetramethylhexadecane	C_20_H_42_	282.55	0.70
22	34.57	1-Hexadecene,7,11,15-trimethyl- 3-methylene-	C_20_H_38_	278.52	1.63
25	35.47	1-Chloro Hexadecane	C_16_H_33_Cl	260.89	0.70
26	35.90	Isocembrol	C_20_H_34_O	290.48	1.18
27	35.97	Methyl hexadecanoate	C_17_H_34_O_2_	270.45	1.25
30	36.58	Palmitic acid	C_16_H_32_O_2_	256.42	1.49
32	36.81	Camphor	C_10_H_16_O	272.00	6.41
33	36.97	Dibutyl phthalate	C_16_H_22_O_4_	278.34	1.33
34	37.14	Ethyl palmitate	C_18_H_36_O_2_	284.48	2.91
35	39.28	Methyl linoleate	C_19_H_34_O_2_	294.47	1.73
36	39.44	Methyl Linolenate	C_19_H_32_O_2_	292.46	0.86
37	39.68	Phytol	C_20_H_40_O	296.53	4.71
38	40.06	Octadecanoic acid 3- octadecyloxypropyl ester	C_39_H_78_O_3_	595.03	0.61
39	40.56	Ethyl Linoleate	C_20_H_36_O_2_	308.50	4.63
40	40.73	Ethyl linolenate	C_20_H_34_O_2_	306.48	2.23
41	40.79	7-Hydroxycoumarine	C_9_H_6_O_3_	162.14	3.54
42	40.93	Osthole	C_15_H_16_O_3_	244.29	8.69
43	42.47	Xanthotoxol	C_11_H_6_O_4_	202.16	1.69

**Table 2:** Experimental design and data for the extraction rate of naringin from the CCD.

No.	A	B	C	Y
1	70	3	20	97.98 ± 0.10
2	70	5	20	98.14 ± 0.07
3	70	4	40	99.74 ± 0.21
4	60	4	20	97.97 ± 0.13
5	60	4	60	98.38 ± 0.11
6	60	5	40	98.67 ± 0.09
7	70	5	60	98.61 ± 0.12
8	80	4	60	98.41 ± 0.15
9	70	3	60	96.95 ± 0.26
10	80	4	20	98.31 ± 0.17
11	70	4	40	99.28 ± 0.14
12	80	3	40	98.27 ± 0.18
13	60	3	40	97.26 ± 0.08
14	70	4	40	99.71 ± 0.11
15	70	4	40	99.50 ± 0.09
16	70	4	40	99.51 ± 0.13
17	80	5	40	98.83 ± 0.20

A = temperature (°C), B = time (h), C = RMG (mesh), Y = extraction rate (%). Results were presented as the mean ± SD (*n* = 3).

**Table 3:** ANOVA for response surface model fitting.

Source	ss	df	ms	F-Value	*p*-Value	Significant
Model	10.08	9	1.12	21.19	0.0003	**
A	0.30	1	0.30	5.62	0.0496	*
B	1.80	1	1.80	34.10	0.0006	**
C	2.420 × 10^−4^	1	2.420 × 10^−4^	4.579 × 10^−3^	0.9479	NS
AB	0.18	1	0.18	3.47	0.1047	NS
AC	0.023	1	0.023	0.43	0.5323	NS
BC	0.56	1	0.56	10.62	0.0139	*
A^2^	0.94	1	0.94	17.87	0.0039	**
B^2^	2.81	1	2.81	53.19	0.0002	**
C^2^	2.74	1	2.74	51.94	0.0002	**
residual	0.37	7	0.053			
lack-of-fit	0.23	3	0.076	2.14	0.2375	NS
pure error	0.14	4	0.035			
the total correction	10.45	16				
R^2^	0.9646					
R_Adj_^2^	0.9191					

**: Statistically significant at *p <* 0.01. *: Statistically significant at *p <* 0.05. NS: Not significant *p >* 0.05.

**Table 4:** Composition and content of IPF pectin and commercially available pectin.

Sample	Total Sugar/%	Galacturonic Acid/%	Protein/%	Ash/%	DE/%
IPF pectin	71.17 ± 0.52 ^a^	59.90 ± 0.79 ^a^	1.93 ± 0.04 ^a^	4.33 ± 0.06 ^a^	44.65 ± 0.02 ^b^
Sigma-Aldrich pectin	77.59 ± 0.32 ^b^	76.98 ± 0.21 ^b^	5.28 ± 0.08 ^b^	8.13 ± 0.04 ^b^	30.45 ± 0.02 ^a^

Results were presented as the mean ± SD (*n* = 3). Different letters indicate statistical significance at *p <* 0.05.

## Data Availability

Not applicable.
